# Cholinergic Denervation Patterns in Parkinson's Disease Associated With Cognitive Impairment Across Domains

**DOI:** 10.1002/hbm.70047

**Published:** 2025-01-23

**Authors:** Emile d'Angremont, Remco Renken, Sygrid van der Zee, Erik F. J. de Vries, Teus van Laar, Iris E. C. Sommer

**Affiliations:** ^1^ Department of Biomedical Sciences of Cells and Systems University Medical Center Groningen Groningen The Netherlands; ^2^ Department of Neurology University Medical Center Groningen Groningen The Netherlands; ^3^ Department of Nuclear Medicine and Molecular Imaging University Medical Center Groningen Groningen The Netherlands

**Keywords:** [^18^F]FEOBV PET, cholinergic degeneration, cognition, Parkinson's disease

## Abstract

Cognitive impairment is considered to be one of the key features of Parkinson's disease (PD), ultimately resulting in PD‐related dementia in approximately 80% of patients over the course of the disease. Several distinct cognitive syndromes of PD have been suggested, driven by different neurotransmitter deficiencies and thus requiring different treatment regimes. In this study, we aimed to identify characteristic brain covariance patterns that reveal how cholinergic denervation is related to PD and to cognitive impairment, focusing on four domains, including attention, executive functioning, memory, and visuospatial cognition. We applied scaled sub‐profile model principal component analysis to reveal cholinergic‐specific disease‐related and cognition‐related covariance patterns using [^18^F]fluoroethoxybenzovesamicol PET imaging. Stepwise logistic regression was applied to predict disease state (PD vs. healthy control). Linear regression models were applied to predict cognitive functioning within the PD group, for each cognitive domain separately. We assessed the performance of the identified patterns with leave‐one‐out cross validation and performed bootstrapping to assess pattern stability. We included 34 PD patients with various levels of cognitive dysfunction and 10 healthy controls, with similar age, sex, and educational level. The disease‐related cholinergic pattern was strongly discriminative (AUC 0.91), and was most prominent in posterior brain regions, with lower tracer uptake in patients compared to controls. We found largely overlapping cholinergic‐specific patterns across cognitive domains, with positive correlations between tracer uptake in the opercular cortex, left dorsolateral prefrontal cortex and posterior cingulate gyrus, among other regions, and attention, executive, and visuospatial functioning. Cross validation showed significant correlations between predicted and measured cognition scores, with the exception of memory. We identified a robust structural covariance pattern for the assessment of cholinergic dysfunction related to PD, as well as overlapping cholinergic patterns related to attentional, executive‐ and visuospatial impairment in PD patients.

## Graphical abstract

In this study, we identified cholinergic brain patterns related to Parkinson's disease and cognitive functioning across several domains, based on the PET tracer [^18^F]FEOBV and SSM/PCA as the method for pattern identification.

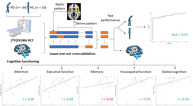

**Summary**

- We identified a Parkinson's disease‐specific cholinergic denervation pattern.
- Cholinergic denervation patterns were associated with attention, executive, and visuospatial functioning, but not memory.
- The cognition‐related patterns showed large overlap in topography, suggesting a cholinergic dependent basic effort for cognitive functioning.

## 1 Introduction

It is well established that besides dopamine also other neurotransmitter systems are seriously implicated in the progression of Parkinson's disease (PD), including the cholinergic system. Cholinergic denervation may occur early in the disease and was found to be more severe in PD‐related dementia than in Alzheimer's disease (Bohnen and Albin 2011; Bohnen et al. 2003). Cholinergic deficiency in PD has been associated with several symptoms, however most prominently with cognitive impairment (Okkels et al. 2024).

Cognitive impairment is considered one of the key features of PD (Aarsland 2016) and largely defines the quality of life of patients (Schrag and Quinn 2000). Cognitive impairment tends to become more prominent in later stages of the disease, but approximately 15%–30% of patients already show mild cognitive impairment at time of diagnosis (Aarsland 2016; van der Zee et al. 2022). Cognitive deficits generally will worsen over time and about 80% of patients ultimately develop Parkinson's dementia (Hely et al. 2008; Aarsland et al. 2003).

The relation between cholinergic dysfunction and cognitive impairment in PD has been established in a number of univariate neuroimaging studies (Hilker et al. 2005; Shimada et al. 2009; Klein et al. 2010; van der Zee et al. 2020). However, univariate analyses treat every voxel or region of interest separately, limiting their ability to convey information about underlying network changes. Previous studies have applied multivariate analysis, using spatial covariance patterns, to identify PD‐related abnormal brain networks (Ma et al. 2007; Teune et al. 2014; Tomše et al. 2017), or brain networks specifically related to cognitive functioning in PD (Mentis et al. 2002; Lozza et al. 2004; Huang et al. 2007; Meles et al. 2015). However, these brain networks were based on brain metabolism and perfusion, assessed with [^18^F]fluorodeoxyglucose positron emission tomography (PET) and MRI, which is not specific to deficits in neurotransmitter activity. Only a few studies assessed covariance patterns in PD in the context of cholinergic (dys)function, and their findings were not cross‐validated (van der Zee, Kanel, Müller, van Laar, and Bohnen, 2022; Colloby et al. 2016). This type of analysis is important to better understand cholinergic dysfunction in PD and how cholinergic dysfunction in PD is related to cognitive decline. Moreover, the identification of covariance patterns allows for individual quantification of cholinergic denervation specifically related to PD and/or cognitive decline, which may have clinical value.

The PET tracer [^18^F]fluoroethoxybenzovesamicol ([^18^F]FEOBV; (−)‐(2R,3R)‐trans‐2‐hydroxy‐3‐(4‐phenylpiperidino)‐5‐(2‐[^18^F]fluoroethoxy)‐1,2,3,4‐tetralin) binds to the vesicular acetylcholine transporter and is a state‐of‐the‐art marker for quantification of brain cholinergic denervation (van der Zee et al. 2019; Okkels et al. 2023a). In this study, we use this PET tracer to identify and cross‐validate cholinergic‐specific spatial covariance patterns related to PD as compared to healthy controls and to identify and cross‐validate cholinergic‐specific patterns related to impairments of specific cognitive domains in patients with PD.

## 2 Methods

### 2.1 Subjects

We included 34 patients (26 men, 8 women, mean age: 67 years, median disease duration: 5 years, clinical diagnosis of PD set by a neurologists) with various levels of cognitive dysfunction and 10 healthy controls (HC; 8 men, 2 women, mean age: 68 years), with similar age, sex, and educational level. Participants were recruited in the context of a separate study aiming to compare cholinergic biomarkers in PD (d'Angremont et al. 2024). Clinical Montreal Cognitive Assessment Scores were monitored before inclusion to ensure a variety in global cognitive functioning within the patient group. Exclusion criteria were the use of cholinesterase inhibitors or drugs with an anticholinergic binding profile, such as clozapine and citalopram (Ehrt et al. 2010; Chew et al. 2008), and the inability to provide informed consent, for example, in case of severe dementia. Other exclusion criteria consisted of contra‐indications for MRI (e.g., ferromagnetic implants) and PET imaging (e.g., pregnancy). For control subjects, an additional exclusion criterion was a history of neurological or neurodegenerative disorders.

The study took place in the University Medical Center Groningen. [^18^F]FEOBV PET imaging and cognitive assessments were aimed to take place within one week. For five participants, there was more than one week, but less than three weeks in between. All participants provided informed consent in accordance with the Declaration of Helsinki. The study was conducted according to the Good Clinical Practice guidelines, approved by the ethical board of the University Medical Center Groningen and registered in the Dutch trial register (NL7838) as part of the study for which the participants were originally recruited.

### 2.2 Cognitive Assessment

Participants underwent a battery of neuropsychological assessments (Table 1). All patients were assessed while using their regular dopaminergic drugs, to reduce the burden of participation. Each neuropsychological assessment was categorized into one of four cognitive domains: memory, attention, executive function, and visuospatial cognition, as shown in Table 1. *Z*‐scores were calculated for all endpoints, relative to the HC group, that is, each individual score was subtracted by the mean of the HC group and divided by the standard deviation (SD) of the HC group. If necessary, the *z*‐scores of a test were inverted (i.e., multiplied by −1) such that a higher score reflected better task performance. We averaged the *z*‐scores of all tests within each domain, creating a composite score, which represented that specific cognitive domain (van der Zee et al. 2020). All domains included at least two neuropsychological tests, as suggested by the recommendations of the Movement Disorder Society (MDS) Task Force (Litvan et al. 2012). An average *z*‐score of the four composite scores was used as a measure of global cognition.

**TABLE 1:** Neuropsychological assessment battery categorized in cognitive domains.

Memory	Attention	Executive function	Visuospatial cognition
Visual Association Test (VAT) Memory Index Score (MIS) of the Montreal Cognitive Assessment	Symbol Digit Modality Test (SDMT) Trail Making Test (TMT) A Stroop Test B	Stroop Test C‐B Trail Making Test (TMT) B Letter fluency	Cube analysis of visual object and space perception battery (VOSP) Map Search of Test of Everyday Attention (TEA) Judgement of Line Orientation (JOLO)

### 2.3 Imaging Acquisition

We obtained an [^18^F]FEOBV PET scan and a T1‐weighted MRI scan of the brain from all subjects. Subjects were injected with approximately 200 MBq of [^18^F]FEOBV, with an allowable range of 180–220 MBq. A low‐dose computed tomography scan for attenuation and scatter correction was performed 210 min after tracer injection, followed by a 30‐min PET scan of the brain, acquired with a Siemens Biograph Vision mCT scanner. PET data was acquired in six 5‐min frames to allow for motion correction. The MR images were obtained on a Siemens 3 Tesla Prisma machine with 1 × 1 × 1.2 mm resolution acquisition and a 64 channel head coil (TR = 2300 ms, TE = 2.98 ms, flip angle = 9°).

### 2.4 Imaging Processing

Image preprocessing was performed using the Statistical Parametric Mapping software package (SPM version 12, Wellcome Trust Center for Neuroimaging). All frames of a PET scan were corrected for motion to the first frame of that scan using a rigid body spatial transformation, after which the frames were averaged. The time‐averaged PET images were co‐registered with the individual T1‐weighted MRI scans, and intensity normalized to a reference region, resulting in parametric standardized uptake value ratio (SUVr) images. For the reference region, we used an eroded version of the supratentorial white matter mask, resulting from a FreeSurfer (http://surfer.nmr.mgh.harvard.edu/) segmentation of the MRI scans, as described elsewhere (Nejad‐Davarani et al. 2019). See Figure S1 for an example. The SUVr images were corrected for partial volume effects (PVE) using the Muller‐Gartner method (Müller‐Gärtner et al. 1992) implemented in the PETPVE12 toolbox in SPM (Gonzalez‐Escamilla et al. 2016). To this end, the anatomical gray matter and white matter fraction were extracted per voxel by applying a second segmentation using the CAT12 toolbox (Gaser et al. 2020). Finally, the images were spatially normalized to MNI space, using the transformation matrix resulting from the CAT12 segmentation, and smoothed using an 8 × 8 × 8 mm kernel.

### 2.5 Pattern Identification

The pattern identification analysis was performed in MATLAB R2020a (Mathworks, Sherborn, MA). We applied scaled sub‐profile modeling based on principal components analysis (SSM/PCA) as a spatial covariance method for pattern identification (Teune et al. 2014; Spetsieris and Eidelberg 2011). SSM/PCA was applied based on the steps described by Spetsieris and Eidelberg (Spetsieris and Eidelberg 2011). Per subject, a mask including only voxels with a value of ≥ 3% of the maximum SUVr value was created. The threshold of 3% was empirically found to generate segmentations that included all cortical gray matter, while most white matter areas were excluded. Hereafter, we created a common mask of voxels that were present in the masks of each individual subject. The common mask was applied to all individual PET images. The SUVr values within the common mask were then log‐transformed. Hereafter, we calculated the mean of the log‐transformed images of the healthy controls, referred to as the group mean profile. The group mean profile was subtracted from each log‐transformed image, such that all voxel values represented the relative difference to the mean of the healthy controls. The resulting images are referred to as subject residual profiles (SRP). As normalization of tracer uptake values was performed in the calculation of SUVr, we did not perform additional within‐subject demeaning. Finally, principal component analysis was applied on the SRP (subjects as rows and voxels as columns). The smallest number of components to explain at least 90% of the total variance were selected for further analysis.

### 2.6 Disease Pattern

A stepwise logistic regression model was applied with disease state (HC or PD) as dependent variable and a linear combination of the principal component scores (i.e., the representation of each subject in the principal component space) as predictors, to identify the cholinergic‐specific disease pattern. The Bayesian information criterion was used for determination of the optimal model. A weighted sum of the model coefficients and the eigenvectors of the corresponding principal components was calculated to form the cholinergic‐specific pattern.

### 2.7 Cognition Patterns

For identification of the cognition patterns, we included only the SRPs of the PD patient group. We re‐applied principal component analysis and selected those components that explained ≥ 90% of variance. Then, stepwise linear regression modeling was applied, using the cognition *z*‐score as dependent variable and a linear combination of the component scores as predictors. This was done separately for each cognitive domain, as well as for global cognition. We again used the Bayesian information criterion to prevent overfitting. Per domain, the cholinergic‐specific pattern was reconstructed by creating a weighted sum of the principal components with their corresponding model coefficients as weights.

It is important to note the difference in representation of the disease pattern and the cognition patterns. The disease pattern was designed to predict whether a subject is a PD patient, with healthy controls as a reference. This means that the pattern represents a PD‐related increase or decrease in tracer uptake, compared to controls. The cognition patterns, on the other hand, were designed to predict individual cognition *z*‐scores. This means that the pattern represents a positive or negative correlation with cognitive domains and overall cognition within the PD group, but not a change in tracer uptake relative to controls.

### 2.8 Pattern Performance Assessment

To assess pattern performance in predicting disease state or cognition scores, we performed leave‐one‐out cross validation. For this purpose, the patterns were derived iteratively with one subject left out. Hereafter, the subject score was calculated for the subject left out, based on the pattern derived from the remaining subjects. A subject score expresses to what extent the identified pattern is present in an individual subject and is calculated by the inner product of the pattern weights and the SRP (i.e., the masked, log‐transformed and centered SUVr image). All voxels within the common mask were used for the subject score calculation. The cross validation included all subjects for validation of the disease pattern and only the PD subjects for validation of the cognition patterns. We assessed the difference in the disease‐related subject scores between patients and healthy controls with a Student's *t*‐test. We also derived the receiver operating characteristic and corresponding area under the curve (AUC) of the cross validation. For all cognition‐related subject scores resulting from cross validation, we performed a Pearson's correlation analysis with each cognition score, assuming a normal distribution in both scores. We considered scores with *p* < 0.05 as significantly different.

To assess the stability of the patterns, we performed bootstrapping on the pattern determination with 5000 repetitions. We subsequently excluded voxels from the original pattern for which the two‐tailed 95% CI of the bootstrap results straddled zero, to establish a pattern including only voxels with a stable non‐zero weight.

## 3 Results

### 3.1 Demographics and Cognition Scores

Table 2 shows the demographics and cognition scores of the included participants. The PD patients scored significantly lower on global cognition and on all assessed cognitive domains, with the exception of memory.

**TABLE 2:** Demographics and cognition scores.

	HC (*n* = 10)	PD (*n* = 34)	*p*
Age (years)	67.6 (8.38)	66.5 (8.31)	0.727
Sex (M/F)	8/2	26/8	1
Disease duration (years; median [IQR])	—	5 [3–7]	—
ISCED (median [IQR])	3 [3–6]	6 [3–6]	0.203
Memory	0 (0.86)	−0.34 (1.38)	0.357
Attention	0 (0.76)	−0.99 (1.96)	**0.021**
Executive function	0 (0.70)	−1.22 (2.72)	**0.023**
Visuospatial cognition	0 (0.61)	−1.65 (2.05)	**< 0.001**
Global cognition	0 (0.60)	−1.08 (1.78)	**0.005**

*Note:* Values are given in mean (SD), unless otherwise indicated. Individual cognition tests were *z*‐scored to the HC group, which is why the composite cognition scores have mean 0 in this group. ISCED: International Standard Classification of Education. ISCED assesses highest level of education, ranging from 0 (early childhood education) to 8 (doctoral or equivalent). Significant values (*p* < 0.05) are shown in bold.

### 3.2 Disease‐Related Pattern

**FIGURE 1:**
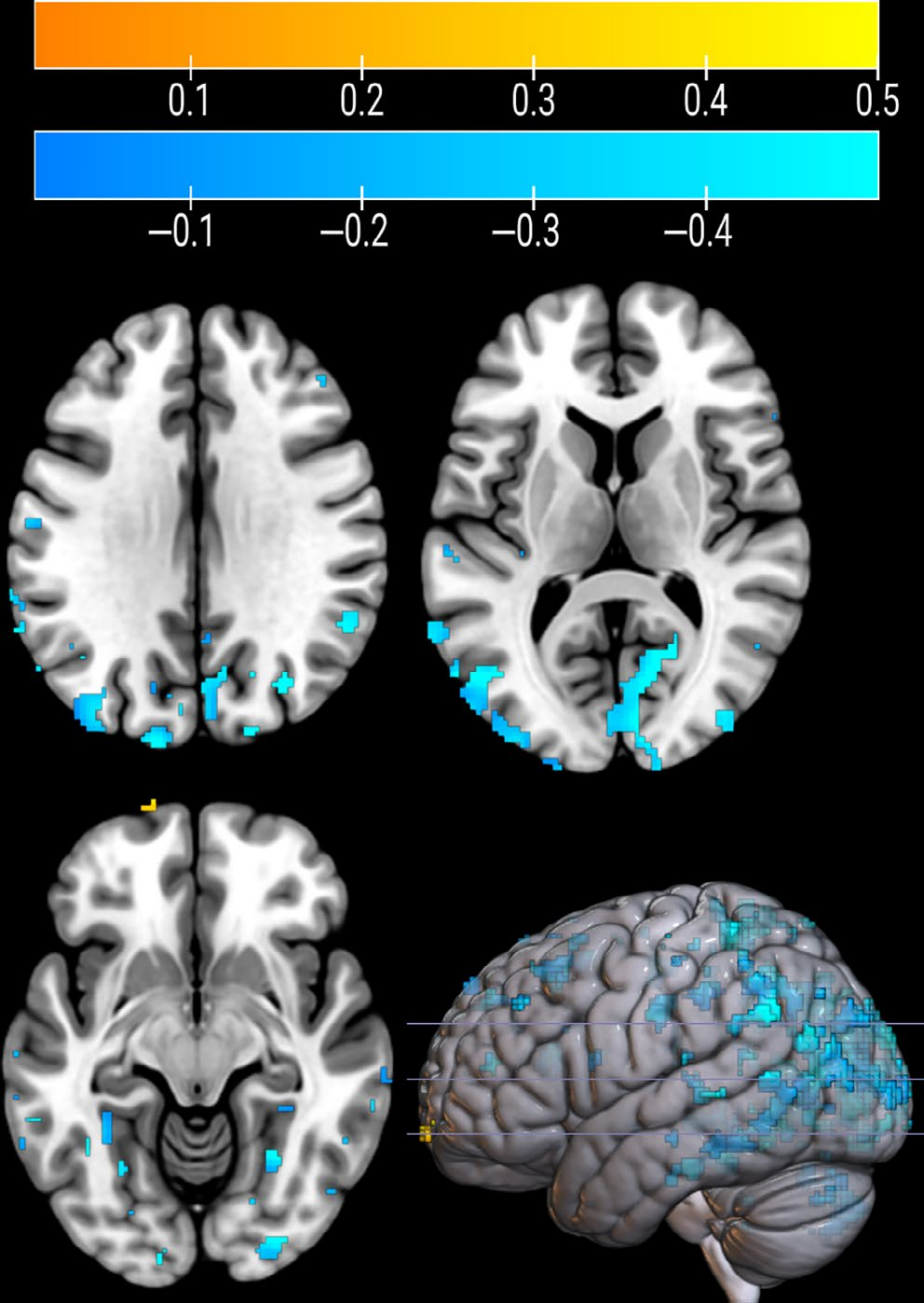
Cholinergic‐specific Parkinson's disease‐related pattern, based on 34 PD patients and 10 healthy controls, after bootstrapping with 5000 repetitions, excluding voxels of which the 95% CI straddled zero. The color represents a positive (red/yellow) or negative (blue) pattern weight, which are interpreted as an increase or decrease in tracer uptake for patients compared to healthy controls, respectively.

Dimensionality reduction of the SRP resulted in 16 principal components, explaining 90.3% of the variance in the data. The optimal model after stepwise logistic regression included four components, together explaining 72.3% of the variance. The resulting disease‐related pattern is shown in Figure S2. We found negative pattern weights (to be interpreted as PD‐related deficits) in posterior regions, including the occipital lobe, inferior parietal lobe, and cerebellum, but also in the lateral frontal gyrus and putamen. After bootstrapping, many clusters of negative weights remained in posterior regions, including the calcarine sulcus, lateral occipital cortex, and superior parietal lobe, as well as the cerebellum (Figure 1). Positive weights, that is, PD‐related increases in tracer uptake, were found in the medial frontal lobe, superior parietal lobe, cingulate gyrus, insula, and caudate nucleus, but only small clusters (< 20 voxels) of positive weights remained in the frontal pole and posterior cingulate gyrus after bootstrapping (Figure 1).

### 3.3 Cognition‐Related Pattern

Dimensionality reduction of the SRP of patients resulted in 12 principal components, explaining 90.5% of the variance in the data. The different cognition patterns included 3 (attentional performance) to 6 (visuospatial functioning) components after stepwise linear regression. The components included in the optimal models together explained a total variance ranging from 71.8% (attention) to 77.7% (global cognition), with the exception of visuospatial functioning (15.6% of variance). Visuospatial functioning was the only domain that did not include the first principal component, which was responsible for 69.8% of the variance.

**FIGURE 2:**
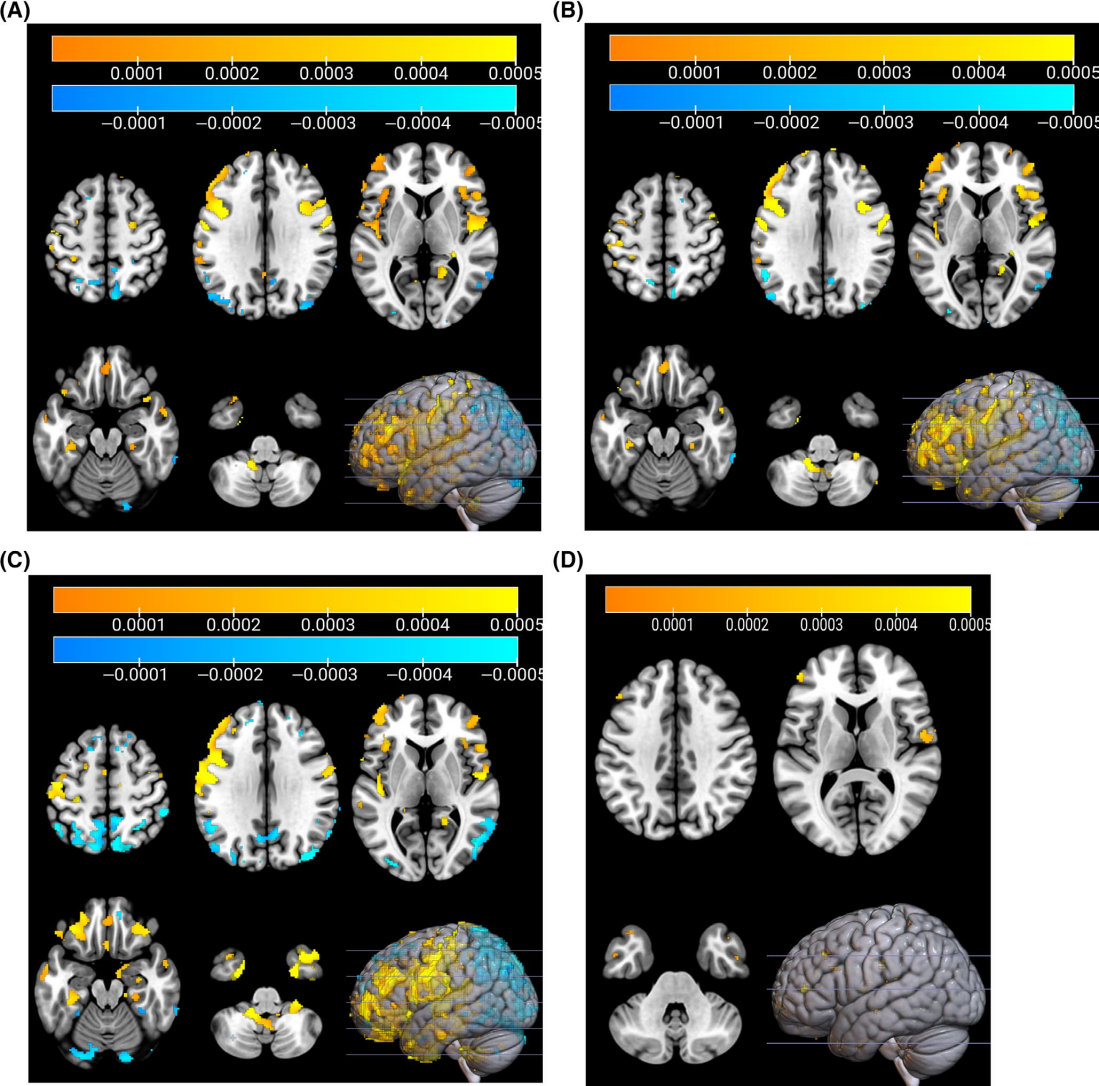
Cholinergic‐specific cognition‐related pattern in Parkinson's disease (*n* = 34), after bootstrapping with 5000 repetitions, excluding voxels of which the 95% CI straddled zero. The color represents a positive (red/yellow) or negative (blue) pattern weight, which are interpreted as a positive or negative correlation between tracer uptake and cognition score, respectively. (A) Attention, (B) executive functioning, (C) visuospatial cognition, (D) memory.

The domains of attention, executive functioning, and visuospatial functioning showed large overlap in their topography. After bootstrapping, positive weights (to be interpreted as a positive correlation between tracer uptake and cognition score) were found in the posterior cingulate gyrus, dorsolateral prefrontal cortex, orbitofrontal cortex, central sulcus, and opercular cortex, among other areas (Figure 2). Negative weights for these domains were primarily found in posterior brain regions. For the memory domain, no negative clusters and very few positive clusters remained after bootstrapping, primarily in the right opercular cortex and left dorsolateral prefrontal cortex (Figure 2D). The cognition‐related patterns before bootstrapping are shown in Figure S3.

### 3.4 Cross Validation

**FIGURE 3:**
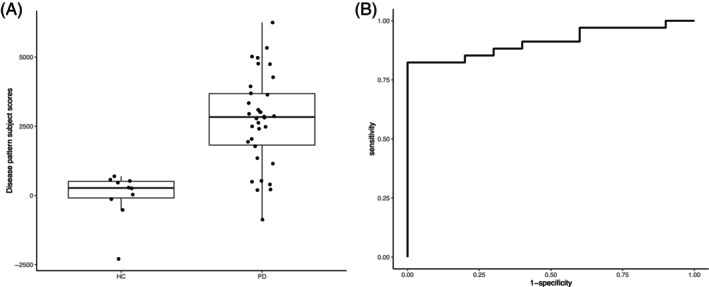
Results of the disease‐related cross validation. Panel A shows the individual subject scores (dots) and boxplot for the healthy controls (left, *n* = 10) and Parkinson's disease patients (right, *n* = 34) (*t* = 6.85, *p* < 0.0001). The subject scores were obtained using a leave‐one‐out cross validation procedure, that is, by applying the obtained disease‐related brain covariance pattern, designed to distinguish patients from controls, to the left‐out subject. Panel B shows the receiver operating characteristic (ROC) curve corresponding to the data presented in panel A (PD vs. controls). The ROC curve had an area under the curve of 0.91.

**FIGURE 4:**
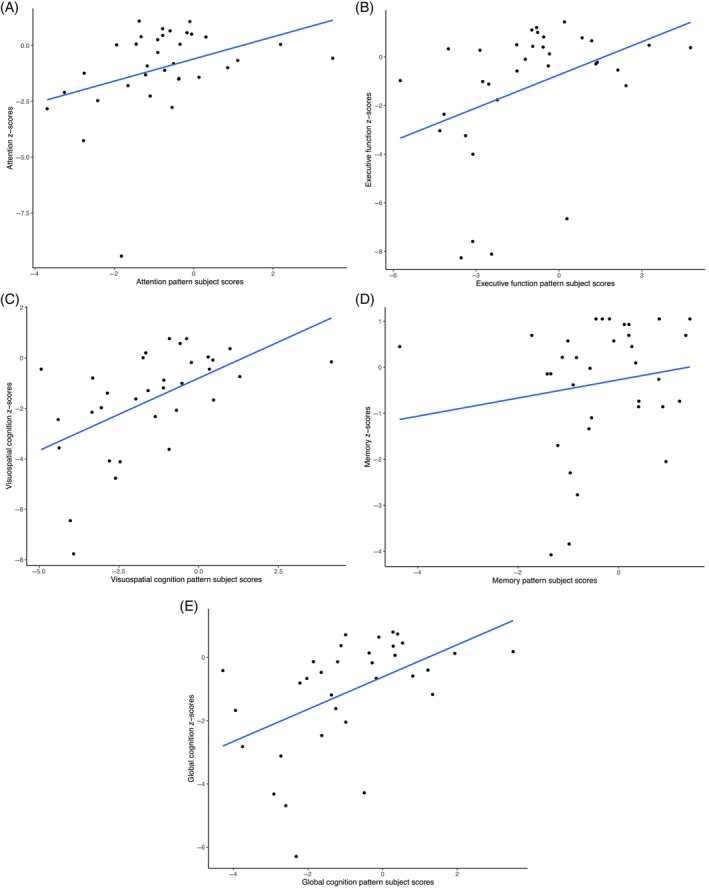
Results of the cognition‐related leave‐one‐out cross validation. Panels (A–E) show the relation between subject scores for all cognitive domains (x‐axis) and the related cognitive performance (y‐axis) in Parkinson's disease patients (*n* = 34); that is, (A) attention (*r* = 0.36, *p* = 0.036), (B) executive functioning (*r* = 0.39, *p* = 0.022), (C) visuospatial cognition (*r* = 0.55, *p* = 0.001), (D) memory (*r* = 0.16, *p* = 0.364), (E) global cognition (*r* = 0.50, *p* = 0.003). Subject scores were obtained using a leave‐one‐out cross validation procedure, that is, by applying the obtained cognition‐related brain covariance pattern, designed to be associated to a specific domain, to the left‐out subject.

In the leave‐one‐out cross validation we iteratively derived patterns on all but one subjects and applied this pattern to the left‐out subject to calculate a subject score. We then compared these subject scores to the ‘true’ values, that is, disease state (PD vs. control) and cognition *z*‐scores across different domains. In this context, a visuospatial cognition pattern subject score is the representation in the left‐out subject of the pattern that has been designed to be related to visuospatial cognition in all remaining subjects The disease‐related subject scores were significantly higher in the PD group, compared to the healthy controls (*t* = −6.85, *p* < 0.0001, Figure 3A). The disease pattern had high discriminative power with an AUC of 0.91 (Figure 3B), resulting in a sensitivity of 82% and a specificity of 100%. The cognition‐related subject scores resulting from cross validation showed significant correlations with the corresponding cognition scores for all domains, except for the memory domain (Table 3, Figure 4).

**TABLE 3:** Correlations of predicted subject scores to composite cognition scores.

Cognition pattern	*r*	*p*
Attention	0.36	**0.036**
Executive function	0.39	**0.022**
Memory	0.16	0.364
Visuospatial cognition	0.55	**0.001**
Global cognition	0.50	**0.003**

*Note:* Significant values (*p* < 0.05) are shown in bold.

In a similar fashion, we also assessed correlations between the disease‐related subject scores of PD patients, designed to predict disease state, and the cognition scores of all domains. This did not provide any significant results (all *p* > 0.1), further demonstrating the specificity of the cognition‐related patterns.

## 4 Discussion

In this study, we identified a robust cholinergic‐specific pattern related to PD, as well as specific cholinergic patterns of several cognitive domains in people with PD. Cross validation showed high discriminative power for the disease‐related pattern, meaning it could be applied to predict disease state (PD vs. control) based on [^18^F]FEOBV tracer uptake with high accuracy. Similarly, significant correlations between the derived and applied cholinergic‐specific cognition patterns and cognition scores on attention, executive‐ and visuospatial functioning, but not memory, were shown. This suggests that these patterns may be applied to predict cognitive functioning in PD, based on [^18^F]FEOBV tracer uptake. The patterns with significant correlations to cognition scores showed a strong overlap in topography.

### 4.1 Cholinergic‐Specific Disease‐Related Pattern

Our cholinergic PD‐related pattern included regions in the posterior parietal and occipital cortices that overlapped with regions found in previous cholinergic imaging studies, which showed cholinergic losses in patients compared to controls, predominantly in posterior brain areas (Shimada et al. 2009; Horsager et al. 2022). The PD‐related deficits in the cerebellum were a surprising result, as cholinergic differences in this brain region are not typically reported in the literature. Nonetheless, decreased acetylcholinesterase activity has been previously found in the cerebellum in PD, specifically related to impaired balance and gait (Gilman et al. 2010). In addition, it should be noted that the pattern weights may be affected by a varying tracer activity in the reference region. Regions with robust PD‐related increases in tracer uptake were extremely small and should thus be interpreted with caution.

The high discriminative power of the disease‐related pattern in cross validation shows that cholinergic degeneration in PD is severe and consistent enough to distinguish patients from controls based on [^18^F]FEOBV tracer uptake. These findings build on previously established loss of cholinergic function in PD (Shimada et al. 2009; Horsager et al. 2022). Additionally, in the context of the relation between cholinergic dysfunction and cognition, it is relevant to have established that the patient group in which we assessed this relationship is indeed distinct from a healthy control group in terms of cholinergic functioning.

### 4.2 Cholinergic‐Specific Patterns of Cognitive Dysfunction

The patterns of attention, executive‐ and visuospatial functioning included regions with a positive correlation between tracer uptake and cognitive functioning in the posterior cingulate, operculum, central sulcus, medial frontal cortex and dorsolateral prefrontal cortex. These findings roughly resemble the differences in [^18^F]FEOBV tracer uptake between dementia with Lewy bodies and PD, showing a more advanced cholinergic denervation in anterior, but not in posterior, brain regions (Okkels et al. 2023b). This suggests that cholinergic terminals in posterior cortical regions are affected early in Lewy body disorders, but that these changes are not sufficient to cause dementia, which is in line with our results suggesting that a decreased tracer uptake specifically in the more anterior regions is related to cognitive decline. Some of these areas, specifically posterior cingulate and medial frontal cortex, overlap with important regions related to the default mode network (DMN). It has been suggested previously that acetylcholine is an important modulator of the DMN (Shah et al. 2016; Hahn et al. 2021). Moreover, a region of interest‐based factor analysis of [^18^F]FEOBV PET in non‐demented PD patients found that the strongest contributor to the association with cognitive functioning strongly overlapped with the DMN (van der Zee, Kanel, Müller, van Laar, and Bohnen, 2022). This component also included pericentral gyri, similar to our findings. The dorsolateral prefrontal cortex, on the other hand, is typically related to executive functioning, as part of the frontoparietal network (Gratwicke, Jahanshahi, and Foltynie 2015). Interestingly, one study found that a preserved muscarinic receptor density in both the DMN and the frontoparietal network was associated with a stronger improvement in Mini‐Mental State Examination (MMSE) score after treatment with cholinesterase inhibitors (Colloby et al. 2016). Future studies should assess whether the cholinergic patterns identified in our study can be useful for treatment prediction.

The negative weights in the overlapping cognition patterns, predominantly found in posterior brain regions, are more difficult to interpret, as a relative decrease of tracer uptake in these areas is related to maintained cognitive functioning. As mentioned, we found PD‐specific deficits in these same regions. One could speculate that a further denervation of cholinergic activity in these areas is associated with a relative preservation in networks that are more strongly related to cognition (van der Zee et al. 2020). Another possible explanation for this unexpected finding is the choice of reference region for our PET analysis. In line with recent literature, we have selected supratentorial white matter as reference. However, cognitive decline in PD may be accompanied by alterations in white matter integrity (Kamagata et al. 2013). It is unknown whether alterations in structural white matter integrity necessarily entail alterations in tracer uptake, but it is possible that our reference region may be differently affected also in terms of tracer uptake in the more cognitively impaired patients compared to the patients with intact cognition. This may be reflected in a negative association in posterior brain regions, as these regions have the most stable cholinergic denervation across patients, as is evident from the PD‐related analysis. Therefore, these findings should be interpreted with caution.

The large overlap in topography in the patterns of attention, executive‐ and visuospatial functioning, suggests that these domains share an underlying cholinergic input. This general involvement of cortical structures across these cognitive domains may also reflect a basic effort, required for all demanding cognitive tasks, which was recently referred to as “cognitive effort investment” (Kührt et al. 2021). Cholinergic neurotransmission may be of vital importance for this cognitive effort investment and, as a result, cholinergic deficits affecting this core function may lead to deficits across multiple cognitive domains, as was seen in this study. In agreement to our results, the mentioned factor analysis, as well as a previously performed voxel‐wise analysis on the same data, showed largely overlapping cholinergic topography across different cognitive domains, including the cingulate cortex, operculum, and insular cortex (van der Zee et al. 2020; van der Zee, Kanel, Müller, van Laar, and Bohnen, 2022). Interestingly, the only exception in these previous studies was the visuospatial domain, whereas in our research we could not establish a stable or predictive pattern for the memory domain. A possible reason for the latter is that memory was not significantly affected in our patient group. A ceiling effect of the included memory tests may play a role in this finding. In addition, there is some evidence that cholinergic deficits may be less involved in memory decline (Bohnen et al. 2005; Calhoun et al. 2004).

The fact that the disease‐related pattern and the cognition‐related patterns showed a distinct topography, together with the finding that the disease‐related subject scores did not correlate with the cognition scores, support the idea that cholinergic dysfunction specific to cognitive impairment is distinct from the cholinergic denervation pattern present in the general PD population. The cognition patterns may, however, be associated with other symptomatology within the patient group, such as motor score, which was not investigated in the current study.

### 4.3 Strengths and Limitations

An important difference between our study and previous cholinergic imaging studies in PD is that most previous work applied a mass‐univariate analysis. Although these analyses are established methods for pinpointing differences or correlations to specific brain areas, these are less sensitive for disease classification and require expert interpretation of the results (Habeck et al. 2008; Carli et al. 2023). The method applied in the current study also takes the covariance between all voxels into account, and provides a more suitable method for the identification of a potential cognitive or disease biomarker.

We categorized our cognitive tests into one of four cognitive domains. However, each test also taps into other domains, making it impossible to create an unique attribution. For example, the Map Search (one of the applied cognition tests, part of the Test of Everyday Attention) was categorized to visuospatial cognition, but also relies heavily on sustained attention.

The application of the current method inherently assumes a certain linearity in disease progression, meaning that at every stage of the disease, the identified patterns will remain constant. In reality these patterns are likely to be dynamic, for example showing regions that may be related to compensatory mechanisms primarily at early stages of the disease, but not at later stages. This is a limitation in the present study, where cognitive functioning was relatively heterogeneous within the patient group. Given a large enough sample size, different patterns may be identified at different disease stages in future studies.

The sample size of our healthy control group was also relatively modest. This may limit the interpretation of the standardized scores in our analysis. However, we are not interpreting absolute values of *z*‐scores, but only in their relation to each other.

Our cross validation showed only weak to moderate correlations between the predicted and measured cognition scores, although statistically significant. Moreover, the patterns in this study were derived from and tested on participants from a single cohort. Validity and generalizability of these patterns needs to be assessed in an independent PD sample.

## 5 Conclusion

We identified a cholinergic PD‐related covariance pattern, as well as several distinct cholinergic cognitive dysfunction patterns in PD. The cognitive dysfunction patterns, specifically related to attention, executive and visuospatial functioning, showed large overlap in topography, suggesting a broad involvement of acetylcholine in all these cognitive domains.

Future studies using an independent cohort should validate whether the identified patterns can serve as a reliable tool to predict efficacy of treatment with cholinesterase inhibitors, for instance in PD patients with (mild) cognitive impairments in specific domains.

## Conflicts of Interest

Dr. T. van Laar has received lecture and/or consultancy fees from AbbVie, Britannia Pharm., Clexio, Centrapharm, Eurocept, Genilec, and Ever Pharma. Dr. E.F.J. de Vries declares financial support from Hoffmann‐La Roche, Eli Lilly, Bristol Myers Squibb, Ionis Pharmaceutical, Rodin Therapeutics, Lysosomal Therapeutics, Novartis, Janssen‐Cilag BV, GE Healthcare and GlaxoSmithKline, for contracted research not related to this study, paid to the institution in the past 5 years. Dr. I. Sommer received speakers fee from Otsuka, is PI of a center that participates in a trial from Boehringer‐Ingelheim and has received a charity grant from Janssen. The other authors have nothing to disclose.

## Supporting information


Data S1.

## Data Availability

The data that support the findings of this study are available on request from the corresponding author. The data are not publicly available due to privacy or ethical restrictions.
