# Supplementary material for: Cholinergic Denervation Patterns in Parkinson's Disease Associated With Cognitive Impairment Across Domains
Source: Hum Brain Mapp. 2025 Jan 23;46(2):e70047. doi: 10.1002/hbm.70047 (PMC11755113; doi:10.1002/hbm.70047)
Supplement: Supplementary file 1 — Data S1. [file HBM-46-e70047-s001.docx]

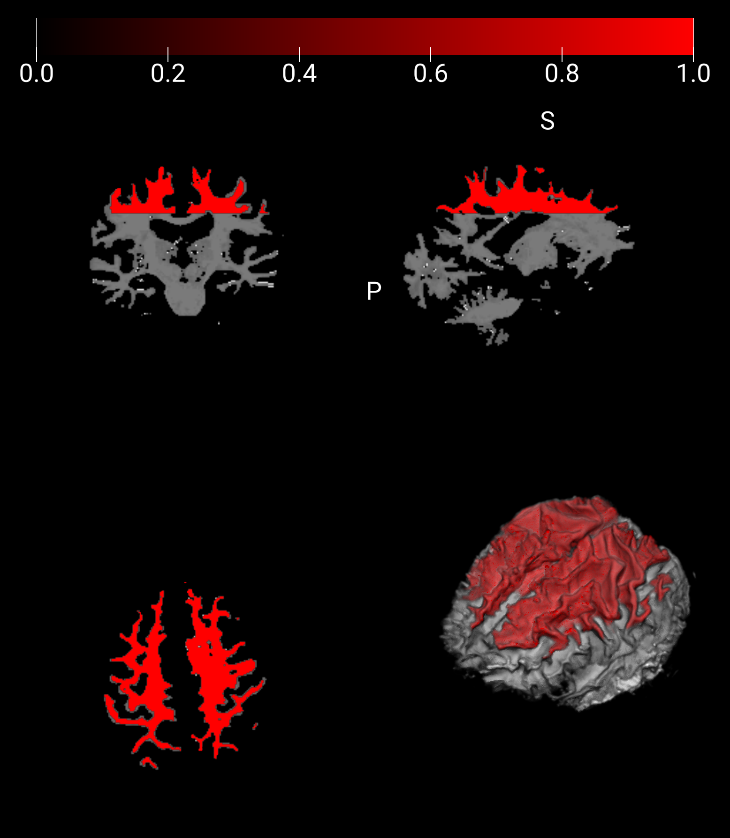


Supplementary Figure 1. Example of eroded supratentorial white matter above lateral ventricles (in red) which was used as reference region to intensity normalize the [^18^F]FEOBV PET images.

**
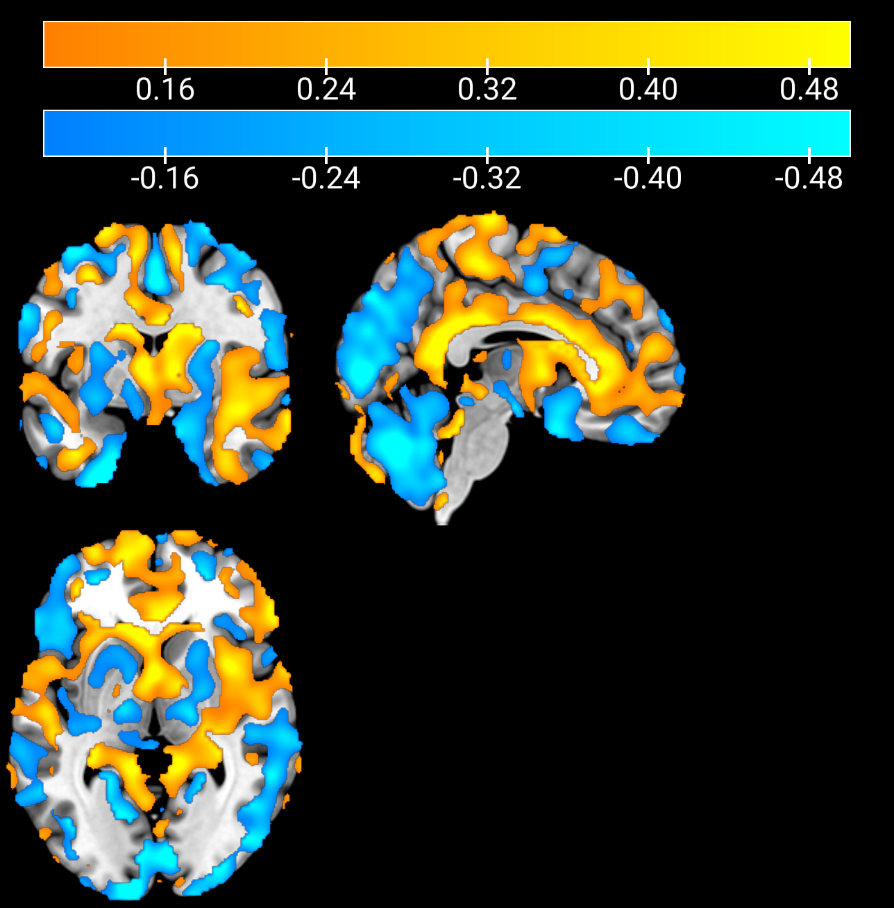
**

Supplementary Figure 2. Cholinergic-specific Parkinson’s disease-related pattern, based on 34 PD patients and 10 healthy controls. The color represents a positive (red/yellow) or negative (blue) pattern weight. Values between -0.1 and 0.1 are not shown.


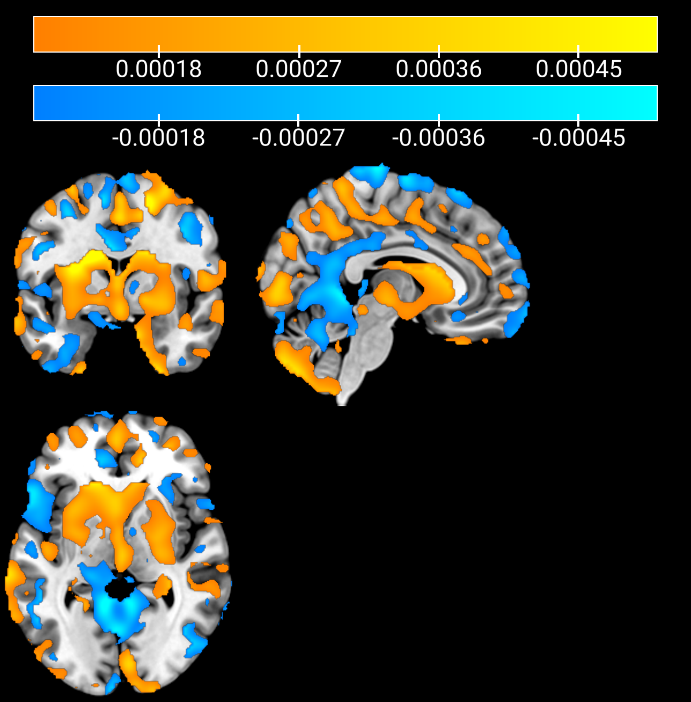

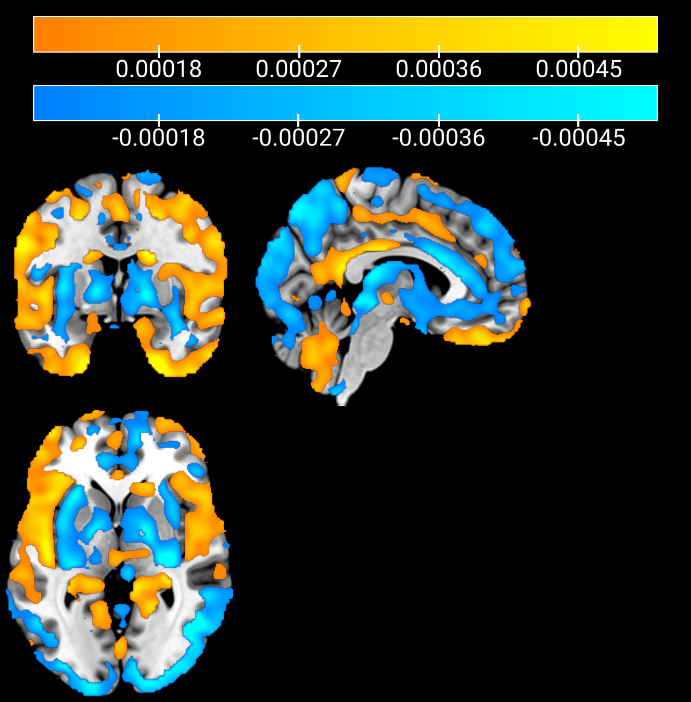

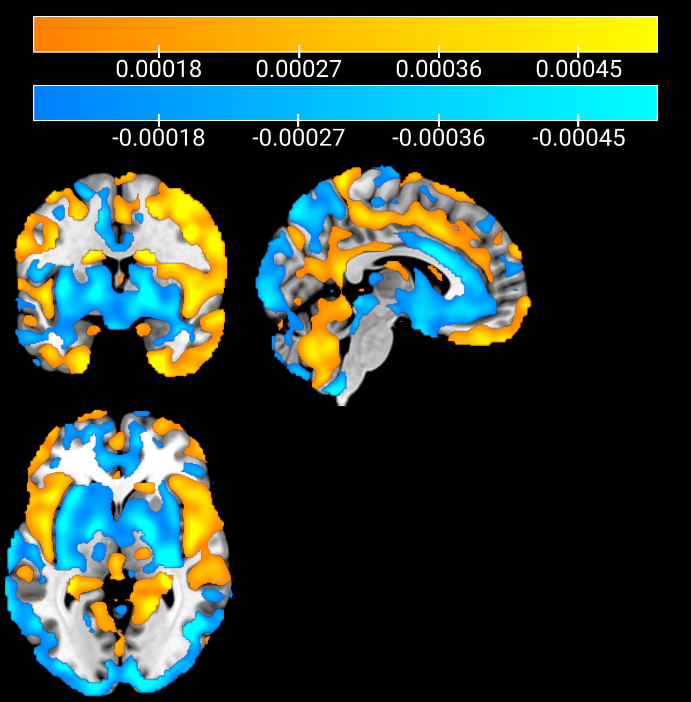

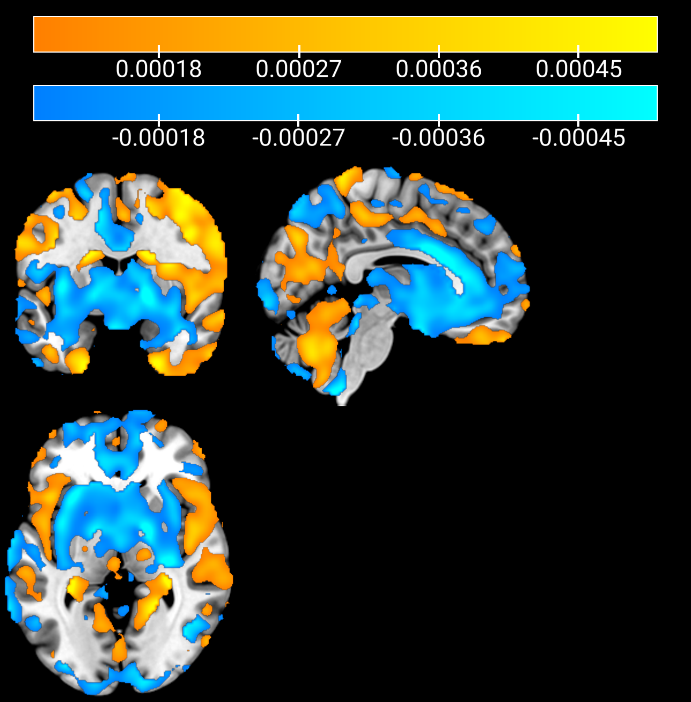


**D**

**C**

**B**

**A**

Supplementary Figure 3. Cholinergic-specific cognition-related patterns in Parkinson’s disease (n=34); A: attention, B: executive functioning, C: visuospatial cognition, D: memory. The color represents a positive (red/yellow) or negative (blue) pattern weight. Values between -0.0001 and 0.0001 are not shown.

**A**

**B**

**C**

**D**

**D**
